# Congenital hereditary endothelial dystrophy with progressive sensorineural deafness (Harboyan syndrome)

**DOI:** 10.1186/1750-1172-3-28

**Published:** 2008-10-15

**Authors:** Julie Desir, Marc Abramowicz

**Affiliations:** 1 Department of Medical Genetics, Hôpital Erasme, ULB, Brussels, Belgium

## Abstract

Harboyan syndrome is a degenerative corneal disorder defined as congenital hereditary endothelial dystrophy (CHED) accompanied by progressive, postlingual sensorineural hearing loss. To date, 24 cases from 11 families of various origin (Asian Indian, South American Indian, Sephardi Jewish, Brazilian Portuguese, Dutch, Gypsy, Moroccan, Dominican) have been reported. More than 50% of the reported cases have been associated with parental consanguinity. The ocular manifestations in Harboyan syndrome include diffuse bilateral corneal edema occurring with severe corneal clouding, blurred vision, visual loss and nystagmus. They are apparent at birth or within the neonatal period and are indistinguishable from those characteristic of the autosomal recessive CHED (CHED2). Hearing deficit in Harboyan is slowly progressive and typically found in patients 10–25 years old. There are no reported cases with prelinglual deafness, however, a significant hearing loss in children as young as 4 years old has been detected by audiometry, suggesting that hearing may be affected earlier, even at birth. Harboyan syndrome is caused by mutations in the *SLC4A11 *gene located at the CHED2 locus on chromosome 20p13-p12, indicating that CHED2 and Harboyan syndrome are allelic disorders. A total of 62 different *SLC4A11 *mutations have been reported in 98 families (92 CHED2 and 6 Harboyan). All reported cases have been consistent with autosomal recessive transmission. Diagnosis is based on clinical criteria, detailed ophthalmological assessment and audiometry. A molecular confirmation of the clinical diagnosis is feasible. A variety of genetic, metabolic, developmental and acquired diseases presenting with clouding of the cornea should be considered in the differential diagnosis (Peters anomaly, sclerocornea, limbal dermoids, congenital glaucoma). Audiometry must be performed to differentiate Harboyan syndrome from CHED2. Autosomal recessive types of CHED (CHED2 and Harboyan syndrome) should carefully be distinguished from the less severe autosomal dominant type CHED1. The ocular abnormalities in patients with Harboyan syndrome may be treated with topical hyperosmolar solutions. However, corneal transplantation (penetrating keratoplasty) represents definitive treatment. Corneal transplantation produces a substantial visual gain and has a relatively good surgical prognosis. Audiometric monitoring should be offered to all patients with CHED2. Hearing aids may be necessary in adolescence.

## Disease name and synonyms

Corneal dystrophy and perceptive deafness (CDPD, OMIM 217400), Corneal dystrophy with progressive sensorineural deafness, Corneal dystrophy and sensorineural prelingual deafness, Harboyan syndrome.

## Background

### The non syndromic endothelial (posterior) corneal dystrophies

The anterior segment of the vertebrate eye is highly specialized and comprises the cornea, trabecular meshwork, iris and lens, whose co-development is essential to normal vision. Amongst these the cornea is the major refracting structure consisting of an anterior stratified epithelium, a paucicellular stroma and an endothelium covering its posterior aspect. The endothelium is a monolayer of polygonal cells that is pivotal to anterior segment development and that maintains corneal transparency by keeping the stroma in a state of relative dehydration [1].

A number of inherited disorders of the cornea have been described in humans [2]. The endothelial (posterior) corneal dystrophies (see Table 1[3-9]), which result from primary endothelial dysfunction, include Fuchs endothelial dystrophy (FECD1 – MIM136800; FECD2 – MIM610158), posterior polymorphous dystrophy (PPCD1 – MIM122000; PPCD2 – MIM609140; PPCD3 – MIM609141) and congenital hereditary endothelial dystrophy (CHED: CHED1 – MIM121700 and CHED2 – MIM217700). They all are thought to represent defects of terminal differentiation of neural crest [10]. This group shares many features including corneal decompensation, altered morphology of endothelial cells, and secretion of an abnormal collagenous layer in the posterior zone of Descemet's membrane (DM), the endothelial basement membrane [11,12].

**Table 1:** The endothelial (posterior) corneal dystrophies – inheritance and onset

	**CHED1**	**CHED2**	**PPCD1**	**PPCD2**	**PPCD3**	**FECD1**	**FECD2**
**Inheritance**	Dominant	Recessive	Dominant	Dominant	Dominant	Dominant	Sporadic or Dominant and more severe in females

**Gene**	20p11.2-q11.2 [3]	*SLC4A11 *[4]	*VSX1 *[5]	*COL8A2 *[6]	*TCF8 *[7]	*COL8A2 *[6]	13pTel-13q12.13 and 18q21.2-q21.32 [8,9]

**Onset**	Early	Birth	Early	Early	Early	Early	Late, 4^th ^decade

FECD is the commonest primary disorder of the corneal endothelium. Signs may be present from the fourth decade of life onwards with the development of focal wart-like guttata in the central cornea arising from Descemet's membrane. The latter is thickened with abnormal collagenous deposition. There is reduced endothelial function and cell density as well as cellular pleomorphism. FECD is usually a sporadic condition but familial, highly penetrant forms showing autosomal dominant inheritance are also recognized [13].

PPCD is a rare bilateral corneal endothelial dystrophy that is inherited in an autosomal dominant manner. The clinical features usually present earlier than FECD, and may be present at birth. The condition is characterized by the formation of blister-like lesions within the corneal endothelium or by regions of endothelial basement membrane thickening with associated corneal oedema. Epithelial-like cells are found in place of the normal amitotic endothelial cells [14], showing abundant intermediate filaments, desmosomes and microvilli [15]. The endothelium becomes multilayered and the abnormally proliferating cells may extend outwards from the cornea over the trabecular meshwork and cause glaucoma.

CHED is believed to result from the hypoplasia or degeneration and dysfunction of the endothelial cells [16]. It can be inherited in an autosomal dominant (CHED1) and an autosomal recessive (CHED2) manner. The endothelium regulates corneal hydration by actively pumping out water from the stroma into the aqueous humor. The Na/K ATPase-driven ion pump plays a crucial role in this mechanism [1]. Excessive water entry into the stroma causes disruption of the collagen fibrils resulting in scattering of light and opacification. Histological features of CHED-affected corneas include diffuse epithelial and stromal edema, defects in the Bowman membrane, paucity of endothelial cells showing degenerative changes *e.g*. multinucleated cells, and a thickened Descemet's membrane reflecting an abnormal secretion by the endothelial cells [17,18].

The autosomal dominant (CHED1) and recessive (CHED2) forms are clinically and genetically distinct [19], and may be distinguished by age at time of onset and by the presence or absence of accompanying signs and symptoms. CHED2 presents at birth or within the neonatal period, while CHED1 usually develops later in childhood. Clinically, CHED2 is generally more severe than CHED1.

Harboyan syndrome is an eye and ear disease, consisting of:

- Congenital hereditary endothelial dystrophy, indistinguishable from CHED2.

- Progressive, postlingual sensorineural hearing loss, mainly affecting the higher frequencies, with first symptoms typically reported in the teenage years.

- All reported cases have been consistent with autosomal recessive inheritance.

## Epidemiology

Population-based epidemiological data for Harboyan syndrome are not available as there are only seven reports of this syndrome in the literature [20-26]. The eleven families reported (24 cases affected) were from various origin (Brazilian Portuguese, Netherlands, Gypsy, Moroccan, Asian Indian, South American Indian, Sephardi Jewish, Dominican). It is of interest that, while more than half of the reported cases were associated with parental consanguinity, several cases resulted from compound heterozygosity (see below), suggesting that carrier frequency may not be extremely low.

Harboyan syndrome appears to be rarer than CHED2. Before the discovery of the gene implicated in the CHED2 etiopathogenesis in 2006, only several cases of CHED2 have been reported. Since then, causative mutations have been detected in 92 CHED2 families originating mainly from regions with a high rate of consanguinity (*e.g*. some regions of India) [4,20,27-33]. As hearing loss in Harboyan syndrome is slowly progressive and may long remain undetected and, at the same time, monitoring of hearing has not been reported in CHED2 patients, it is possible that some cases of Harboyan syndrome are currently reported as nonsyndromic CHED2.

## Clinical description

**Figure 1:**
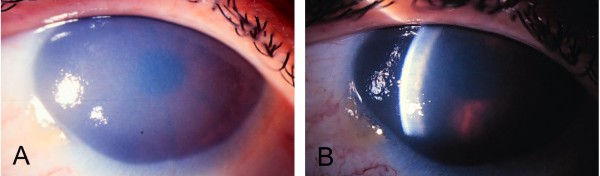
**Eye phenotype, untreated adult with Harboyan syndrome**. The cornea presents congenitally with a ground glass, bluish-white opaque cornea from diffuse edema of the stroma (1A). Slit lamp examination showing milkiness and increased thickness of the corneal (1B).

CHED presents as a ground glass, bluish-white opaque cornea (Figure 1A). It is due to diffuse edema of the stroma resulting from endothelial cell dysfunction [16] and leads to visual loss (the presence of an otherwise normal anterior segment).

Harboyan syndrome, similarly to CHED2, manifests as a diffuse, bilateral corneal edema, with a "ground glass cornea" appearance. Corneal clouding is observed at birth or within the neonatal period, with minimal progression over time. The most frequent additional sign is nystagmus, which is presumably caused by the severe corneal clouding present from early in life.

**Figure 2:**
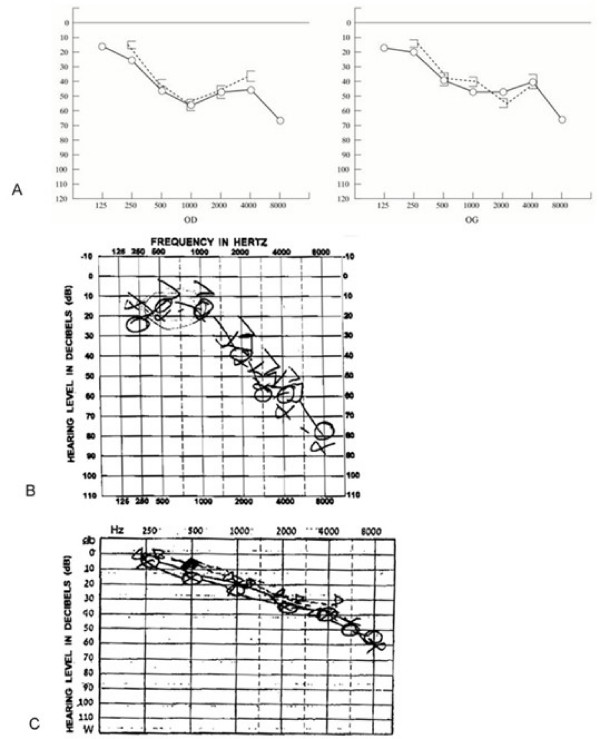
**Sensorineural hearing loss in Harboyan syndrome**. Typical hearing loss in three Harboyan patients. Hearing deficit is in the 20–50 db range (mild to moderate), mainly affecting the higher frequencies. 2A: Patient 1 at 33 years (right and left ear). 2B: Patient 2 at 18 years. 2C: Patient 3 at 19 years.

Hearing loss in Harboyan syndrome is not reported at birth, and no case of prelingual deafness has been reported so far. It is sensorineural, slowly progressive, with typical deficits in the 20–50 db range (mild to moderate) at ages 10–25 yrs, and mainly affects the higher frequencies (Figure 2). Although symptomatic hearing loss is not reported in early childhood, it might probably be detected in the first years of life if sought [24]. In families with Harboyan syndrome, hearing loss (when investigated) has been found as early as age 4 years in all subjects with CHED [20].

## Etiology

### Genetics

In 1995, Toma performed genetic linkage analysis with microsatellite markers on a seven generation British CHED1 pedigree, and mapped CHED1 to a 2.7cM region of chromosome 20p11.2-q11.2 with a multipont lod score of 9.34 between D20S48 and D20S471 [3]. This 2.7 cM region lied within the 30 cM region linked to posterior polymorphous dystrophy (PPD). PPD is genetically heterogeneous, and PPD at 20p11 has been associated with mutations in the paired-like homeodomain gene *VSX1 *[5]. The CHED1 gene remains unknown.

In 1999, a large, consanguineous Irish pedigree with CHED2, was linked to chromosome 20p13 with a maximum lod score of 9.30 at microsatellite marker D20S482. The critical region of homozygosity spanned 8 cM between markers D20S113 and D20S882. Mapping data clearly indicated that the autosomal recessive CHED2 gene was distinct from the autosomal dominant CHED1 gene [34].

Harboyan syndrome was also mapped to 20p13 in one large consanguineous Moroccan family between markers D20S199 and D20S437 with a maximum multipoint lod of 4.20 at D20S889/D20S179 [26]. The locus was named CDPD1. The critical 7.73 cM linkage region overlapped the linkage region of autosomal recessive CHED2.

In 2006, analysis of the *SLC4A11 *gene located within the linkage overlap, in CHED2 patients showed seven different mutations in ten families. Mutations were predicted to cause loss of protein function either by impeding membrane targeting or by nonsense-mediated decay [4]. The study of 3 consanguineous and 3 nonconsanguineous families with Harboyan syndrome revealed homozygosity or compound heterozygosity, respectively, for *SLC4A11 *mutations in Harboyan patients, indicating that CHED2 and Harboyan syndrome are allelic disorders [20]. A total of 62 different *SLC4A11 *mutations have been reported in 98 families (92 CHED2 and 6 Harboyan) [4,20,27-33]. Roughly the same proportion of truncating and missense mutations have been observed in both disorders, with no obvious clustering of mutations. Of note, two residues were found mutated in both CHED2 and Harboyan patients. The first case was deletion 473_480del8bp. The predicted protein change was however slightly different in the two reports: Arg158GlnfsX4 in CHED2 [31], and Arg158ProfsX4 in Harboyan [20], because of a single nucleotide polymorphism (SNP, rs3827075) at the nucleotide immediately following the deletion, which was A/A in the CHED2 patient and C/C in the Harboyan patient. In the second case, the same residue, Serine 213, was involved in two different missense mutations: c.638C→T (Ser213Leu) in a CHED2 patient [25], and c.637T→C (Ser213Pro) in a Harboyan patient [20]. The latter Harboyan patient was a compound heterozygote, and her second mutation (Met856Val) might theoretically explain the different phenotype. It remains possible that some cases reported as CHED2 consisted in fact of unrecognised Harboyan cases in whom hearing loss had not yet developed, or had been overlooked.

Heterozygous *SLC4A11 *mutations have recently been reported in Fuchs endothelial corneal dystrophy (FECD), a late-onset progressive disorder of the corneal endothelium, indicating that carriers parents of affected CHED2 or Harboyan children might be at risk of developing late-onset corneal dystrophy [35]. Association of Fuchs and either CHED or Harboyan syndrome in the same family has however not been reported so far.

### Pathophysiology

*SLC4A11 *encodes a ubiquitous electrogenic sodium-coupled borate transporter (also called BTR1 or NABC1) which is essential for cellular boron homeostasis, and whose defect hampers cell growth and proliferation [36]. *SLC4A11 *is related to the SLC4 family of transport proteins, which consists of a functionally diverse group of 11 members that play an essential role in the transport of HCO3- [37]. Although *SLC4A11 *is a highly selective boron concentrating transporter, in boron-free medium, it functions as a Na+ and OH- permeable channel [36]. CHED2 was the first human disease associated with a boron transporter defect, although boron homeostasis remains poorly understood in humans. Consistent with *SLC4A11 *mutations as a cause of hearing loss, this gene is expressed in the cochlea of adult mice, more specifically at the level of the lateral wall, which contains the stria vascularis [38], the latter being involved in the highly distinctive homeostasis of cochlear fluid and endolymph secretion. Considering the highly specific constitution of the endolymph and the expression of the gene in the stria vascularis [38], a role of *SLC4A11 *in Na+ and OH- homeostasis in the inner ear cannot be excluded. Comparative SAGE analysis of gene expression profiles showed that *SLC4A11 *is downregulated in Fuchs endothelial dystrophy [39]. *In situ *hybridization showed expression of *SLC4A11 *in the mouse cornea at embryonic day 18, which corresponds to human gestational month 5, the time at which CHED pathology is believed to develop in humans [4]. *SLC4A11 *is expressed in human corneal endothelium as shown by reverse transcriptase polymerase chain reaction (RT-PCR) [4].

## Diagnostic methods

The diagnosis of CHED is based upon clinical criteria and detailed ophthalmological assessment. All cases of autosomal recessive CHED (CHED2) and all cases of Harboyan syndrome reported so far have shown mutations of, or have been consistent with linkage to *SLC4A11*, with no evidence of genetic heterogeneity. A molecular confirmation of the clinical diagnosis is hence feasible.

### Ophthalmological assessment

Harboyan as well as CHED2 patients present with bilateral clouding of the entire cornea appearing within the first years of life. Central corneal thickness is increased. Slit lamp examination shows diffuse opacification with epithelial and stromal edema (Figure 1B). Visual acuity is usually severely affected and nystagmus may be present. The endothelial barrier function can be assessed by fluoro-photometrical method. Endothelial dystrophy can be confirmed by the histopathologic findings of the explanted cornea that show severely degenerated corneal endothelial cells and abnormal thickening of Descemet's membrane [18].

### Audiometry

Tonal audiometry in patients with Harboyan syndrome shows sensorineural hearing loss. Hearing loss is postlingual, slowly progressive, with a variable age of onset, ranging from 4 to 19 years in the patients studied to date, and a variable degree of hearing loss, -30 dB to -60 dB, in one study of six Harboyan families with various ethnic backgrounds [20].

## Differential diagnosis

The differential diagnosis between Harboyan syndrome and CHED2 relies on the audiometry examination data, showing hearing loss in Harboyan syndrome; otherwise, both conditions share the same ocular abnormalities.

Harboyan syndrome and CHED2 differ from CHED1. In CHED1, opacification is not present at birth and is usually seen after the first or second year of life. In contrast to Harboyan syndrome and CHED2, accompanying signs such as photophobia and epiphora are common presentation of CHED1 (these signs taper with the progression of the corneal clouding), but nystagmus is rarely observed.

A variety of genetic, metabolic, developmental, acquired, and cryptogenic causes can result in congenital clouding of the cornea. Congenital corneal opacities present in approximately 3/100,000 newborns [40]. In a study by Rezende *et al *[41], among 47 cases of congenital corneal abnormalities, the first cause was Peters anomaly (40%), followed by sclerocornea (18%), dermoid (15%), congenital glaucoma (7%), microphthalmia (4%), birth trauma, and metabolic disease (3%). Seven cases (9%) were classified as idiopathic. Ten patients had systemic abnormalities associated with their ocular condition.

Peters anomaly is not an isolated anterior segment abnormality. Rather, it occurs as a variable, phenotypically heterogeneous condition associated with several underlying ocular and systemic defects. Central, paracentral, or complete corneal opacity is always present in patients with Peters anomaly. Blood vessels are typically not found within the opaque portion of the cornea. This feature is helpful to distinguish Peters anomaly from other causes of congenital corneal opacity.

Sclerocornea is an uncommon developmental abnormality of the anterior segment due to mesenchymal dysgenesis. It is usually seen as an isolated ocular abnormality involving both eyes, although it can occur unilaterally. This condition typically occurs sporadically but may also have a familial or autosomal dominant inheritance pattern. On clinical evaluation, patients with partial sclerocornea have a peripheral, white, vascularized, 1- to 2-mm corneal rim that blends with the sclera, obliterating the limbus. The central cornea is generally normal. In total sclerocornea, the entire cornea is involved, but the center of the cornea is clearer than the periphery. This finding distinguishes it from Peters anomaly, in which the center is most opaque. The opacification affects the full thickness of the stroma and hampers visualization of the posterior corneal surface and of the intraocular structures (Figure 1B). Histopathology reveals disorganized collagenous tissue containing fibrils that are larger than normal. Other findings may be present which include a shallow anterior chamber, abnormalities of the iris and the lens, and microphthalmos. Systemic abnormalities, such as limb deformities and craniofacial and genitourinary defects, can also accompany this finding.

Limbal dermoids are benign congenital tumors that contain choristomatous tissue (tissue not normally found at that site). They most frequently appear at the inferior temporal quadrant of the corneal limbus. However, they are occasionally present entirely within the cornea or confined to the conjunctiva. Inheritance is usually sporadic, although autosomal recessive or sex-linked pedigrees exist. They can be associated with corneal clouding. Although most limbal dermoids are isolated findings, approximately 30% are associated with Goldenhar syndrome, especially when they are bilateral.

Congenital glaucoma is an important cause of congenital corneal clouding. CHED itself may cause glaucoma, and a clear association between congenital glaucoma and congenital hereditary endothelial dystrophy has been described. CHED should hence be suspected where persistent and total corneal opacification fails to resolve after normalization of intraocular pressure [42,43].

## Genetic counseling

A genetic counseling is recommended in all cases of CHED. The recurrence risk is 25% in siblings of both CHED2 and Harboyan syndrome, with no symptoms reported in heterozygous carriers. Care must be taken to differentiate the autosomal recessive types of CHED (CHED2 and Harboyan syndrome) from the autosomal dominant CHED1. In the absence of a clear clinical history, careful examination of relatives, *SLC4A11 *gene analysis, and clinical presentation, should allow to distinguish the usually less severe CHED1, where deafness has not been reported [17].

## Management

Topical hyperosmolar solutions (hyertonic sodium chloride) produce temporary corneal dehydration and may be beneficial in some patients.

Corneal transplantation (penetrating keratoplasty) is the definitive treatment. It is recommended to avoid amblyopia and to restore vision. Penetrating keratoplasty carries a relatively good surgical prognosis and can produce a substantial visual gain even when carried out late in life. Postoperatively, patients should expect only gradual recovery of vision. Following surgery, the best vision may not be obtained after six to twelve months, or more.

Careful audiometry is recommended in all cases of CHED2. Audiometric monitoring of hearing is advisable because of non-congenital, progressive onset of hearing loss in Harboyan syndrome, with some cases requiring hearing aids.

## Prognosis

All patients with Harboyan syndrome described to date are in good general health, with no other systemic features that reduce life expectancy. Corneal transplantation carries a relatively good surgical prognosis and can produce a substantial visual gain even when carried out late in life. The disease has not been reported to recur after corneal transplantation. In the absence of longitudinal data, the hearing prognosis is presently unclear in the very young, sporadic CHED patients.

## List of abbreviations

CHED: Congenital hereditary endothelial dystrophy of the cornea; CDPD: Corneal dystrophy with progressive sensorineural deafness; PPCD: Posterior polymorphous corneal dystrophy; FECD: Fuchs endothelial corneal dystrophy

## Competing interests

The authors declare that they have no competing interests.

## Authors' contributions

The authors equally contributed to this review article. They read and approved the final version of the manuscript.
